# Prediction of major adverse cardiovascular events following ST-segment elevation myocardial infarction using cardiac obesity marker—epicardial adipose tissue mass index: a prospective cohort study

**DOI:** 10.3389/fcvm.2025.1539500

**Published:** 2025-02-12

**Authors:** Zeyan Liu, Jinbo Wang, Yanfang Yang, Jinglin Cheng, Min Yang, Ye Zhang

**Affiliations:** ^1^ Department of Emergency Internal Medicine, Chest Pain Center (CPC), Second Affiliated Hospital of Anhui Medical University, Hefei, China; ^2^ Department of Radiology, Second Affiliated Hospital of Anhui Medical University, Hefei, China; ^3^ Department of Intensive Care Unit II, Second Affiliated Hospital of Anhui Medical University, Hefei, China; ^4^ Department of Anesthesiology & Perioperative Medicine, Second Affiliated Hospital of Anhui Medical University, Hefei, China

**Keywords:** ST-segment elevation myocardial infarction, epicardial adipose tissue, major adverse cardiovascular events, prediction, obesity

## Abstract

**Background:**

Although reperfusion therapy has led to improvements in the acute phase of ST-segment elevation myocardial infarction (STEMI), the incidence of major adverse cardiovascular events (MACE) following STEMI has not significantly decreased. The accumulation of epicardial adipose tissue (EAT) may be associated with poorer STEMI prognosis and could serve as a potential prognostic marker. However, research examining this relationship remains limited.

**Methods:**

This single-center prospective study enrolled 308 STEMI patients. Patients were randomly assigned to training set and validation set in a 7:3 ratio. The primary outcome was MACE one-year post-STEMI. Epicardial adipose tissue mass index (EAMI) was calculated as EAT volume divided by absolute value of the EAT attenuation index, measured using coronary computed tomography angiography (CTA). The relationship between EAMI and MACE was analyzed using Kaplan–Meier curves, Cox regression, and restricted cubic spline (RCS) plots. The predictive performance of EAMI was assessed through receiver operating characteristic (ROC) curves, C-index, net reclassification index (NRI), integrated discriminant improvement (IDI), coefficient of determination (R^2^), calibration curves, Brier score, and decision curve analysis (DCA) with comparisons to the GRACE score. Subgroup analyses were conducted based on age, gender, body mass index (BMI), left ventricular ejection fraction (LVEF), and culprit artery.

**Results:**

A total of 308 patients were included in the analysis, with 212 in the training set and 96 in the validation set. In the training set, Kaplan–Meier survival analysis revealed that higher EAMI levels were associated with an increased cumulative risk of MACE. Cox multivariate regression analysis indicated that EAMI was independently associated with MACE (HR = 2.349, 95% CI 1.770–3.177, *P* < 0.001). Restricted cubic spline (RCS) analysis suggested a positive dose-response relationship between EAMI and MACE (*P* for nonlinearity = 0.87). EAMI showed better discriminative ability, prediction effect, accuracy, and clinical applicability compared to the traditional GRACE score. In the validation set, EAMI also demonstrated good predictive performance for MACE. Subgroup analyses suggested that EAMI's predictive ability was consistent across various demographic and clinical characteristics.

**Conclusion:**

EAMI has high value in predicting MACE in patients 1-year after STEMI, helps identify high-risk patients with poor prognosis in early clinical practice.

## Introduction

Acute myocardial infarction (AMI) is a cardiovascular emergency resulting from coronary artery obstruction, leading to myocardial necrosis. According to the characteristics of electrocardiogram, it is divided into ST-segment elevation myocardial infarction (STEMI) and non ST-segment elevation myocardial infarction (NSTEMI), STEMI basically represents transmural necrosis of the myocardium. Epidemiological data show that approximately 7 million people are affected by AMI annually worldwide, with 500,000 new cases in China each year (1–3). While the success of acute-phase reperfusion has markedly reduced in-hospital mortality, the incidence of post-discharge major adverse cardiovascular events (MACE) remains largely unchanged (2).

Obesity is a key prognostic factor in AMI, with 11.98% of AMI-related cardiovascular deaths attributed to high BMI (4). However, BMI, as a systemic indicator, is not accurate enough in evaluating cardiac obesity, and even leads to completely opposite erroneous evaluations. Epicardial adipose tissue (EAT) is anatomically adjacent to the myocardium, and its release of lipophilic cytokines continues to affect myocardial cells. Especially for damaged myocardium, EAT can further exacerbate the damage. Compared to BMI, EAT is currently considered a more powerful indicator for evaluating cardiac obesity. Research indicates that excessive EAT accumulation contributes to myocardial inflammation, fibrosis, and cell apoptosis, leading to ventricular remodeling and heart failure (5, 6). Moreover, EAT continuously releases inflammatory factors that act on myocardial tissue and has a long-lasting effect, making it suitable as a predictive indicator for medium and long-term MACE. Compared with the serological indicator troponin I and the ultrasound indicator ejection fraction, EAT has stronger stability and does not show significant changes in the short term. A single measurement after admission can reflect the cardiac fat deposition over a period of time. In terms of clinical research, the higher the prevalence of EAT in STEMI patients, the larger the infarct size (IS) and coronary microvascular occlusion (MVO) (7, 8). EAT may disrupt myocardial electrophysiology, influencing the development of arrhythmias (9). Thus, EAT accumulation could be a significant factor in STEMI prognosis, warranting further exploration. In terms of measurement methods, previous studies have certain limitations. Ultrasound can only measure thickness and cannot accurately reflect EAT volume. Although cardiac magnetic resonance imaging (CMR) or enhanced computed tomography (CT) can accurately measure volume, researchers have often overlooked the simultaneous assessment of the degree of inflammatory response in EAT.

Although most studies on EAT and cardiovascular diseases focus on stable coronary disease and atrial fibrillation, research specifically addressing EAT in STEMI is limited. Early identification of high-risk STEMI patients is critical for improving treatment outcomes. While MACE prediction post-STEMI has relied more on cardiac itself structural and functional indicators, EAT, through its direct interaction with the myocardium, may influence post-STEMI myocardial remodeling. Current research on EAT in this context is sparse, with most metrics focusing on volume, and limited attention paid to fat density or attenuation index. This study aimed to assess STEMI prognosis using EAT imaging parameters, particularly the epicardial adipose tissue mass index (EAMI), which combines epicardial adipose tissue volume (EATV) and epicardial adipose tissue attenuation index (EAAI). Considering the pathological effects of EAT on myocardium, EAMI may hold potential as a reliable indicator for predicting major cardiovascular adverse events following STEMI.

## Methods

### Study population

**Figure 1:**
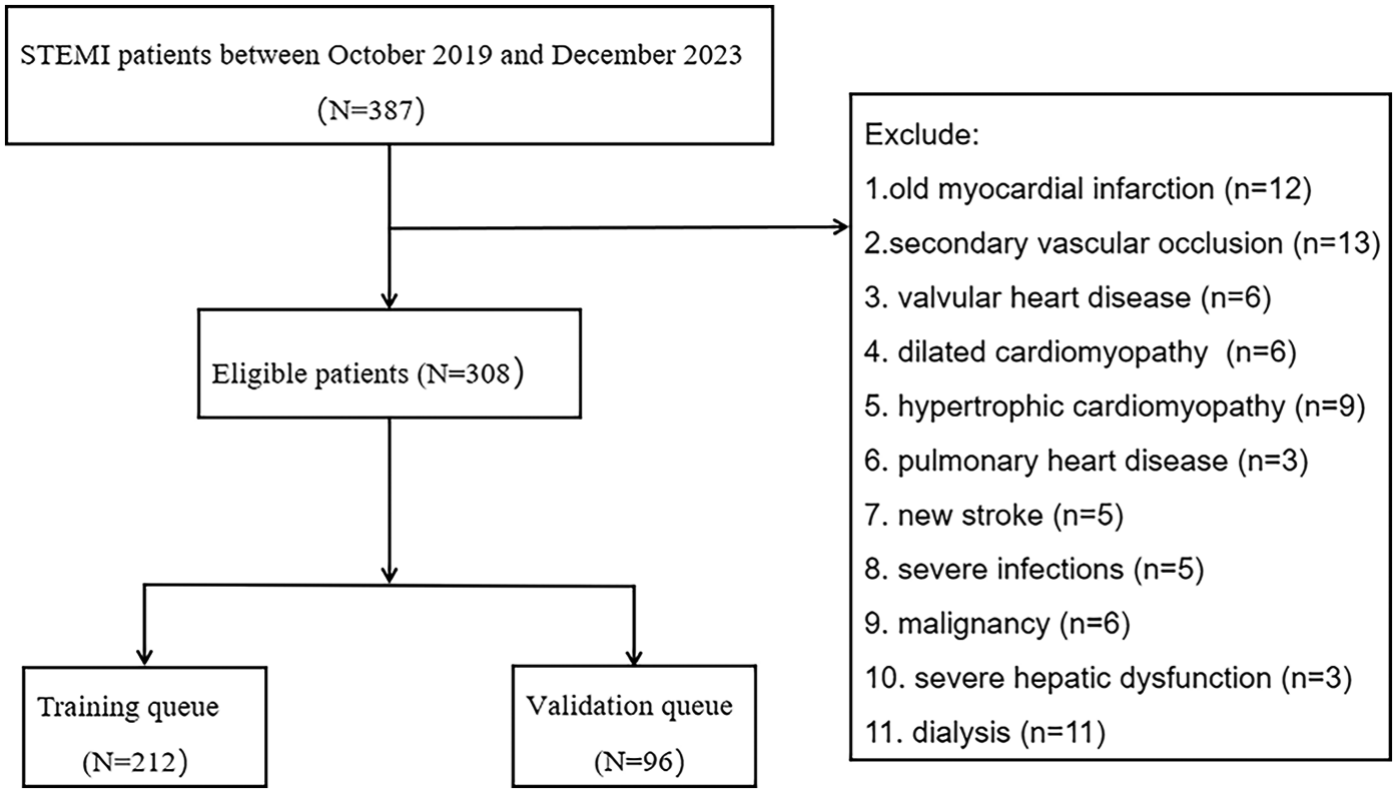
Study flow chart.

This study followed the principles of the Helsinki Declaration and adhered to the Strengthening the Reporting of Observational Studies in Epidemiology (STROBE). The Ethics Committee of the Second Affiliated Hospital of Anhui Medical University approved this study (Approval Number: YX2022-001). Moreover, all included patients in this study obtained informed consent from the patients themselves or authorized family members. The inclusion criteria were as follows: (1) patients treated at the Second Affiliated Hospital of Anhui Medical University between October 2019 and December 2023; (2) diagnosis of AMI based on the fourth edition of the universal definition of myocardial infarction (10); (3) confirmed ST-elevation myocardial infarction (STEMI) by electrocardiogram; (4) age between 18 and 90 years; (5) successful reperfusion within 24 h, with a door-to-wire time (D2W) of less than 90 min. Exclusion criteria included: (1) previous myocardial infarction; (2) secondary vascular occlusion; (3) valvular heart disease, dilated cardiomyopathy, hypertrophic cardiomyopathy, or pulmonary heart disease; (4) new onset stroke; (5) severe infection; (6) malignancy; (7) severe liver dysfunction or dialysis (Figure 1).

Sample size estimation was based on previous reports of the one-year incidence of MACE incidence rate in STEMI patients within 1-year post-discharge. Specifically, we referenced the study by Aldujeli et al. (11), which reported a one-year MACE incidence of 20.95% in STEMI patients. Using this incidence rate, we applied a standard sample size calculation formula for cohort studies, assuming a two-sided *α* = 0.05 and *β* = 0.20 (80% power), and a 10% estimated dropout rate. Based on these parameters, the minimum required sample size for this cohort was calculated to be 200 patients.

### Data collection

Baseline data were collected through preliminary literature review and expert consultations, including demographic, clinical, laboratory, body composition, and echocardiographic parameters. The Global Registry of Acute Coronary Events (GRACE) score was recorded as a comparative indicator, and the culprit artery was used as a subgroup identifier. Demographic data, clinical parameters, laboratory measurements, and GRACE score were collected prior to reperfusion, while echocardiographic data were obtained within 3 days after reperfusion.

The baseline variables included: age, gender, body mass index (BMI), smoking history, alcohol consumption, co-morbidities of hypertension and diabetes mellitus, symptom onset time, door-to-wire (D2W) time, Killip classification, hemoglobin (HGB), platelet count (PLT), platelet distribution width (PDW), liver enzymes [alanine aminotransferase (ALT), aspartate aminotransferase (AST)], creatinine (Cr), total bilirubin (TBIL), N-terminal pro B-type natriuretic peptide (NT-proBNP), high-sensitivity troponin I (hs-CTnI), myoglobin (MYO), creatine kinase MB (CK-MB), glycosylated hemoglobin (HbA1c), uric acid (UA), triglycerides (TG), low-density lipoprotein cholesterol (LDL-c), high-density lipoprotein cholesterol (HDL-c), lipoprotein a (Lpa), homocysteine (HCY), D-dimer, fibrinogen degradation product (FDP), C-reactive protein (CRP), neutrophil-lymphocyte ratio (NLR), left ventricular ejection fraction (LVEF), left ventricular end-diastolic volume (LVEDV), left ventricular end-systolic volume (LVESV), left ventricular diastolic function (LVDF), interventricular septal thickness (IVS), left ventricular posterior wall thickness (LVPW), intraoperative slow-reflow/no-reflow, intraoperative hypotension, intraoperative ventricular arrhythmias, final angiographic TIMI (thrombolysis in myocardial infarction) flow grade, and the SYNTAX (SYNergy between PCI with TAXUS™ and Cardiac Surgery) score.

Considering the large range of data, hs-CnTI, MYO, CKMB and NT-proBNP are convert to ordinal variables based on their median values. BMI, LVEF, LVDF, LVESV, and LVEDV are categorized into ordinal groups according to established guidelines or consensus (12–14). The culprit artery was recorded as the left anterior descending artery (LAD), left circumflex artery (LCX), or right coronary artery (RCA), with LCX and RCA grouped as non-LAD.

### EAT imaging

**Figure 2:**
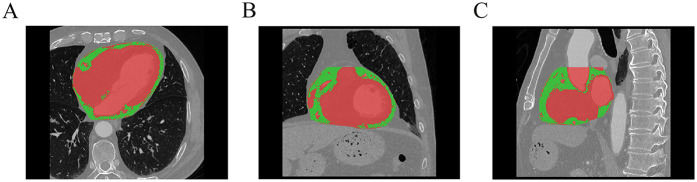
Schematic diagram of EAT delineation and measurement (**A**: axial, **B**: coronal, **C**: sagittal, green part indicate the EAT, Red part indicate the myocardium and blood vessels).

All enrolled patients underwent coronary computed tomography angiography (CCTA) within 5 days after reperfusion, performed using either a Philips Brilliance iCT or Philips Spectral IQon scanner. The scan range extended from 1 cm below the tracheal bifurcation to the base of the heart. Three-dimensional reconstruction was performed from the original CCTA images. A semi-automated quantitative method was employed to segment the adipose tissue within the pericardial silhouette, specifically targeting regions with CT values ranging from −190 to −30 HU, to ensure accurate analysis (15). The extracted adipose tissue was further processed to remove outliers and out-of-range voxels. EATV and EAAI were measured using a Siemens post-processing workstation (syngo.via) (Figure 2), and EAMI was calculated as the ratio of EATV to the absolute value of EAAI. All procedures were conducted by two senior radiologists who were blinded to the study's objectives and clinical data.

### Outcomes processing

Patients were regularly followed up through outpatient visits or telephone consultations. Efforts were made to minimize loss to follow-up. In cases of loss to follow-up, the last available data were included in the analysis to maintain data integrity.

The primary endpoint was the occurrence of MACE, including recurrent AMI, heart failure with reduced ejection fraction (HFrEF), stroke and all-cause mortality. Among them, recurrent AMI was defined as the recurrence of AMI after hospitalization, which may involve restenosis of the original lesion or the development of new coronary artery lesions. The diagnosis of HFrEF was made in accordance with the 2022 AHA/ACC/HFSA Heart Failure Management Guidelines (14).

### Statistical analysis

The STEMI patients included in the analysis were randomly divided into a training set and a validation set in a 7:3 ratio (16). Multiple imputation was applied to address missing baseline data, with missing values accounting for less than 5%. Data were presented as mean ± standard deviation, median (interquartile range) or frequency (%), as appropriate.

The cumulative incidence rate of MACE was estimated using the Kaplan–Meier method and differences between the high and low EAMI groups were compared using the log-rank test. The optimal cut-off value for dichotomizing EAMI was determined using the surv-cutpoint function of the survminer package in R language. Univariable and multivariable Cox proportional hazards regression analyses were conducted to assess the prognostic value of various parameters for MACE and identify independent predictors (To avoid collinearity, EATV and EAAI, as components of EAMI, are not included in the multivariate Cox analysis). A restricted cubic spline analysis was performed to examine the dose-response relationship between EAMI and MACE risk.

To evaluate the prognostic value of EAMI, its performance was compared to the GRACE score using metrics including the area under the ROC curve (AUC), concordance index (C-index), net reclassification index (NRI), and integrated discriminant improvement (IDI). Comparison between AUCs using *z*-test. The goodness of fit (accuracy) was assessed using coefficient of determination (R^2^), Brier score and calibration curves. Additionally, the clinical utility was evaluated using decision curve analysis (DCA).

Finally, patients were stratified into subgroups based on age (<65 years or ≥65 years), gender (male or female), BMI (<28 kg/m^2^ or ≥28 kg/m^2^), LVEF (<50% or ≥50%) (14), and culprit artery (LAD or Non-LAD). Cox regression and C-index analyses were used to assess the impact and predictive power of EAMI on MACE within each subgroup.

Statistical analyses were conducted using SPSS (version 26.0) and R (version 4.2.1). A two-sided *P*-value < 0.05 was considered statistically significant.

## Results

### Baseline patient characteristics

A total of 308 patients were included in the analysis after applying the inclusion and exclusion criteria. The training set consisted of 212 STEMI patients, with 80.7% male and a mean age of 58.02 years, while 10 patients were lost to follow-up. Among these, 45 patients experienced major adverse cardiovascular events (MACE), with the frequency of adverse events in the following order: HFrEF, recurrent AMI, stroke, and death. The validation set included 96 STEMI patients, with 6 lost to follow-up and 21 patients experiencing MACE. Baseline characteristics of both the training and validation cohorts were comparable (Table 1).

**Table 1:** Balance test of baseline data between training set and validation set.

Variables	Total (*n* = 308)	Training (*n* = 212)	Validation (*n* = 96)	*P*
EAMI, *n* (%)				0.723
<1.49 cm^3^/Hu	149 (48.4)	104 (49.1)	45 (46.9)	
≥1.49 cm^3^/Hu	159 (51.6)	108 (50.9)	51 (53.1)	
Grace score, Median (Q1,Q3)	129.50 (110.00, 156.00)	126.00 (108.00, 154.25)	132.00 (112.00, 162.00)	0.138
Age, *n* (%)				
<65 years	209 (67.9)	143 (67.5)	66 (68.8)	0.821
≥65 years	99 (32.1)	69 (32.5)	30 (31.2)	
Gender, *n* (%)				0.761
Male	247 (80.2)	171 (80.7)	76 (79.2)	
Female	61 (19.8)	41 (19.3)	20 (20.8)	
D-to-W Time, Median (Q1,Q3), min	76.00 (63.00, 96.00)	75.50 (63.00, 96.00)	77.00 (63.75, 94.25)	0.817
BMI, *n* (%)				
<18.5 kg/m^2^	4 (1.3)	3 (1.4)	1 (1.0)	0.672
18.5–24 kg/m^2^	121 (39.3)	85 (40.1)	36 (37.5)	
24–28 kg/m^2^	117 (38.0)	81 (38.2)	36 (37.5)	
≥28 kg/m^2^	66 (21.4)	43 (20.3)	23 (24.0)	
Smoking, *n* (%)				0.451
No	167 (54.2)	118 (55.7)	49 (51.0)	
Yes	141 (45.8)	94 (44.3)	47 (49.0)	
Drinking, *n* (%)				0.363
No	275 (89.3)	187 (88.2)	88 (91.7)	
Yes	33 (10.7)	25 (11.8)	8 (8.3)	
Onset time, Median (Q1,Q3), hours	12.00 (8.00, 16.00)	12.00 (8.00, 16.00)	13.00 (8.00, 17.25)	0.394
Hypertension history, *n* (%)				0.943
No	121 (39.3)	83 (39.2)	38 (39.6)	
Yes	187 (60.7)	129 (60.8)	58 (60.4)	
Diabetes history, *n* (%)				0.106
No	209 (67.9)	150 (70.8)	59 (61.5)	
Yes	99 (32.1)	62 (29.2)	37 (38.5)	
NLR, Median (Q1,Q3), %	3.97 (2.36, 8.08)	3.66 (2.33, 7.74)	5.06 (2.56, 8.70)	0.073
CRP, Median (Q1,Q3), ug/ml	2.20 (0.50, 8.40)	2.05 (0.50, 6.65)	2.60 (0.50, 9.85)	0.324
HGB, Median (Q1,Q3), g/L	145.00 (131.00, 155.00)	145.00 (132.00, 153.00)	144.50 (117.50, 162.25)	0.878
PLT, Mean ± SD, × 10^9^/L	217.03 ± 69.99	216.55 ± 68.30	218.10 ± 73.97	0.861
PDW, Median (Q1,Q3), %	14.25 (11.57, 16.30)	15.00 (11.80, 16.30)	13.65 (10.00, 16.20)	0.115
AST, Median (Q1,Q3), U/L	39.00 (24.00, 150.25)	33.00 (22.75, 114.25)	33.50 (22.00, 122.50)	0.277
ALT, Median (Q1,Q3), U/L	35.00 (25.00, 62.25)	34.00 (25.00, 56.00)	38.50 (27.75, 77.75)	0.215
TBIL, Median (Q1,Q3), umol/L	12.50 (8.60, 17.25)	12.50 (8.70, 16.92)	12.30 (8.20, 18.27)	0.901
UA, Median (Q1,Q3), umol/L	342.00 (270.00, 396.25)	343.00 (274.00, 396.25)	329.50 (267.50, 398.25)	0.534
NT-proBNP, Median (Q1,Q3), pg/ml	276.50 (67.75, 765.50)	234.00 (54.75, 688.25)	238.50 (56.75, 709.50)	0.615
Hs-CTnI, Median (Q1,Q3), pg/ml	2,362.00 (235.15, 23,083.25)	2,180.00 (149.65, 24,604.97)	2,719.00 (641.88, 19,430.00)	0.187
CKMB, Median (Q1,Q3), U/L	17.91 (8.84, 47.96)	17.91 (9.43, 43.35)	18.12 (5.70, 57.83)	0.743
HB1AC, Median (Q1,Q3), %	5.80 (5.50, 6.50)	5.80 (5.50, 6.40)	5.80 (5.50, 6.85)	0.357
TG, Median (Q1,Q3), mmol/L	1.42 (0.95, 2.15)	1.41 (0.95, 2.15)	1.44 (0.94, 2.15)	0.743
HDL, Median (Q1,Q3), mmol/L	1.19 (1.00, 1.37)	1.18 (1.00, 1.36)	1.25 (1.00, 1.39)	0.645
LDL, Median (Q1,Q3), mmol/L	3.04 (2.43, 3.52)	3.00 (2.33, 3.50)	3.08 (2.51, 3.63)	0.213
HCY, Median (Q1,Q3), mmol/L	15.40 (12.38, 20.40)	15.15 (12.50, 19.85)	16.55 (10.60, 21.85)	0.961
LVEF, *n* (%)				
≥50%	250 (81.2)	173 (81.6)	77 (80.2)	0.688
40%-49%	35 (11.4)	23 (10.8)	12 (12.5)	
<40%	23 (7.4)	16 (7.5)	7 (7.3)	
LVESV, *n* (%)				0.301
Male 15–62 ml, female 13–47 ml	236 (76.6)	166 (78.3)	70 (72.9)	
Male >62 ml, female >47 ml	72 (23.4)	46 (21.7)	26 (27.1)	
LVEDV, *n* (%)				0.725
Male 53–156 ml, female 46–121 ml	260 (84.4)	180 (84.9)	80 (83.3)	
Male >156 ml, female >121 ml	48 (15.6)	32 (15.1)	16 (16.7)	
IVS, Median (Q1,Q3), mm	10.00 (9.00, 11.00)	10.00 (9.00, 11.00)	10.00 (8.00, 11.25)	0.176
LVPW, Median (Q1,Q3), mm	9.00 (8.00, 10.00)	9.00 (9.00, 10.00)	9.00 (8.00, 10.00)	0.569
LVDF, *n* (%)				0.206
0	43 (14.0)	35 (16.5)	8 (8.3)	
I	128 (41.6)	89 (42.0)	39 (40.6)	
II	88 (28.6)	57 (26.9)	31 (32.3)	
III	49 (15.9)	31 (14.6)	18 (18.8)	
No reflow slow blood flow, *n* (%)				0.182
No	221 (71.8)	157 (74.1)	64 (66.7)	
Yes	87 (28.2)	55 (25.9)	32 (33.3)	
Intraoperative hypotension, *n* (%)				0.248
No	264 (85.7)	185 (87.3)	79 (82.3)	
Yes	44 (14.3)	27 (12.7)	17 (17.7)	
Intraoperative ventricular arrhythmia, *n* (%)				0.496
No	272 (88.3)	189 (89.2)	83 (86.5)	
Yes	36 (11.7)	23 (10.8)	13 (13.5)	
TIMI blood flow classification, *n* (%)				0.496
No	272 (88.3)	189 (89.2)	83 (86.5)	
Yes	36 (11.7)	23 (10.8)	13 (13.5)	
SYNTAX score, Median (Q1,Q3)	20.00 (16.00, 25.00)	20.25 (16.00, 25.00)	19.00 (16.00, 23.12)	0.157
Culprit artery, *n* (%)				0.935
LAD	140 (45.5)	95 (44.8)	45 (46.9)	
LCX	64 (20.8)	45 (21.2)	19 (19.8)	
RCA	104 (33.8)	72 (34.0)	32 (33.3)	

EAMI, epicardial adipose tissue mass index; GRACE, Global Registry of Acute Coronary Events; BMI, body mass index; NLR, Neutrophil-lymphocyte ratio; CRP, C-reactive protein; HGB, hemoglobin; PLT, platelet count; PDW, platelet distribution width; ALT, alanine aminotransferase; AST, aspartate aminotransferase; Cr, creatinine; TBIL, total bilirubin; UA,uric acid; NT-proBNP, N-terminal pro B-type natriuretic peptide; MYO, myoglobin; Hs-CTnI, high-sensitivity troponin I; CK-MB, creatine kinase MB; FDP, fibrinogen degradation product; HbA1c, glycosylated hemoglobin; TG, triglycerides; HDL-c, high-density lipoprotein cholesterol; LDL-c, low-density lipoprotein cholesterol; Lpa, lipoprotein a; HCY, homocysteine; LVEF, left ventricular ejection fraction; LVEDV, left ventricular end-diastolic volume; LVESV, left ventricular end-systolic volume, LVDF, left ventricular diastolic function; IVS, interventricular septal thickness; LVPW, left ventricular posterior wall thickness; TIMI, thrombolysis in myocardial infarction; SYNTAX, SYNergy between PCI with TAXUS™ and Cardiac Surgery; LAD, left anterior descending artery; LCX, left circumflex artery; RCA, right coronary artery.

### Kaplan–Meier curve

**Figure 3:**
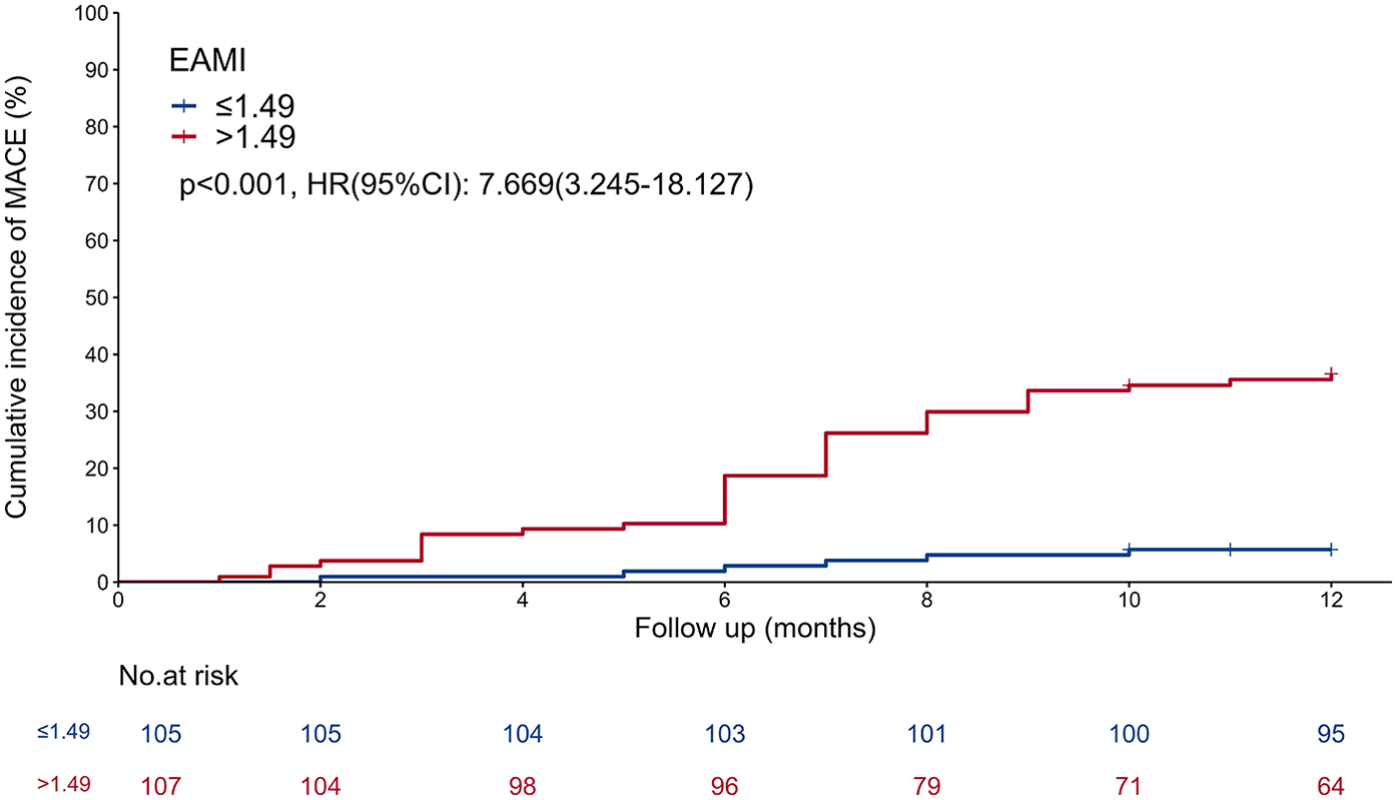
Kaplan–meier curve showing comparison of the cumulative incidence of MACE between the EAMI ≤ 1.49 cm^3^/Hu group and the EAMI > 1.49 cm^3^/Hu group.

In the training set, patients were classified into high- and low-EAMI groups based on an optimal cutoff value of 1.49 cm^3^/Hu. The Kaplan–Meier curve (Figure 3) demonstrated a significantly higher cumulative incidence of MACE in the high-EAMI group compared to the low-EAMI group (*P* < 0.001).

### Relationship between EAMI and MACE

**Figure 4:**
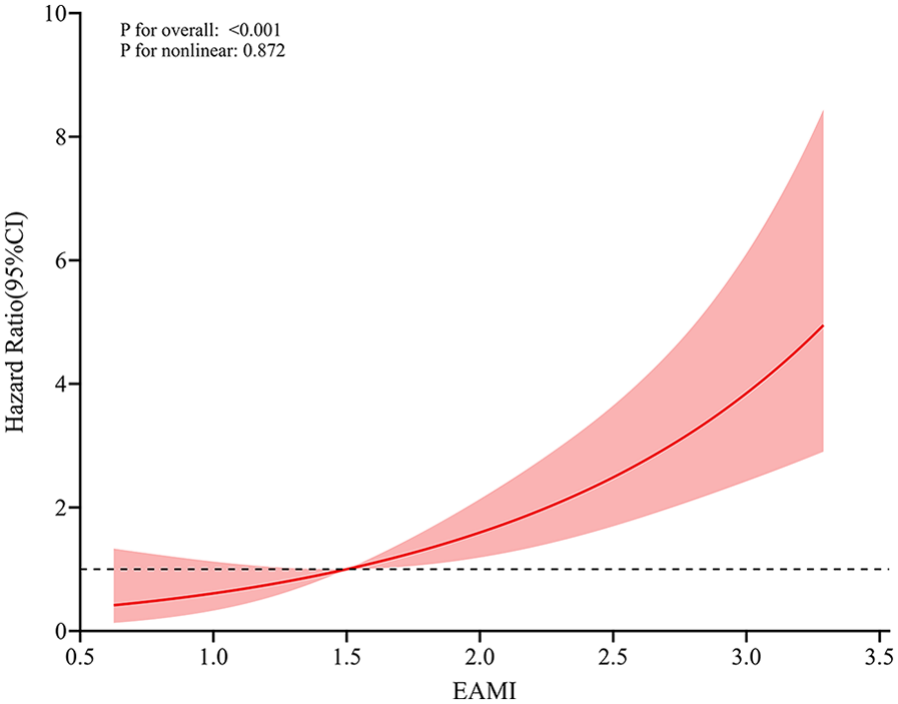
RCS analysis for the dose-response relationship between EAMI and MACE.

In training set, univariable Cox regression analysis (Table 2) showed that EAMI, EATV, EAAI, age, killip classification, LVEF, LVEDV, NLR, AST, Cr, TBIL, UA, NT-proBNP, hs-CTnI and CK-MB were all significantly associated with MACE. Multivariable Cox regression analysis (Table 2) indicated that EAMI (HR = 2.349, 95% CI 1.770–3.177, *P* < 0.001) was an independent risk factor for MACE after adjusting for confounders. Furthermore, restricted cubic spline analysis revealed a linear dose-response relationship between EAMI and MACE (Figure 4, *P* for nonlinearity = 0.872).

**Table 2:** Univariable and multivariable Cox regression analysis for MACE.

	Univariable analysis		Multivariable analysis	
Variables	HR (95% CI)	*P*	HR (95% CI)	*P*
EAMI（>1.49 vs. ≤1.49 cm^3^/Hu）	2.659 (1.727, 4.094)	**<0.001**	2.349 (1.770, 3.177)	**<0.001**
EATV（>116 vs. ≤116 cm^3^）	1.108 (1.009, 1.026)	**<0.001**	–	–
EAAI（> −76.9 vs. ≤−76.9 Hu）	1.041 (1.024, 1.059)	**<0.001**	–	–
Gender (Female vs. Male)	0.903 (0.420, 1.939)	0.793		
Age (≥65 vs. <65 years)	1.048 (1.023, 1.075)	**<0.001**	1.031 (1.006, 1.057)	**0.017**
D-to-W Time (min)	1.000 (0.992, 1.008)	0.921		
Killip classification（II vs. I）	1.987 (1.289, 3.077)	**0.002**	0.853 (0.505, 1.441)	0.552
Killip classification（III vs. I）	2.685 (1.577, 4.733)	**<0.001**	1.420 (1.219, 2.124)	**0.008**
Killip classification（IV vs. I）	6.124 (3.274, 12.065)	**<0.001**	1.635 (1.156, 2.276)	**0.005**
BMI（18.5–24.0 vs. <18.5 kg/m^2^）	0.809 (0.107, 6.098)	0.837		
BMI（24.0–28.0 vs. <18.5 kg/m^2^）	0.867 (0.115, 6.541)	0.890		
BMI（≥28.0 vs. <18.5 kg/m^2^）	0.954 (0.121, 7.529)	0.964		
Smoking history (yes vs. no)	0.991 (0.550, 1.784)	0.975		
Drinking history (yes vs. no)	0.936 (0.370, 2.373)	0.890		
Hypertension (yes vs. no)	0.663 (0.370, 1.190)	0.168		
Diabetes mellitus (yes vs. no)	1.565 (0.857, 2.860)	0.145		
Onset time (hour)	1.023 (0.969, 1.081)	0.411		
NLR (%)	1.068 (1.019, 1.119)	**0.006**	1.018 (0.956, 1.085)	0.599
CRP (ug/ml)	1.002 (0.989, 1.016)	0.728		
HGB (g/L)	0.986 (0.969, 1.002)	0.093		
PLT (×10^9^/L）	0.996 (0.991, 1.000)	0.063		
PDW (%)	0.978 (0.903, 1.060)	0.591		
ALT (U/L)	1.001 (1.000, 1.001)	**0.010**	1.000 (0.999, 1.000)	0.450
AST (U/L)	1.002 (0.996, 1.008)	0.551		
Cr (umol/L)	1.003 (1.001, 1.005)	**0.025**		
TBIL (umol/L)	1.011 (1.003, 1.019)	**0.007**	1.003 (0.993, 1.014)	0.575
UA (umol/L)	1.001 (1.000, 1.001)	**0.026**	1.001 (1.000, 1.002)	**0.003**
NT-proBNP (>234 vs. ≤234 pg/ml)	4.447 (2.141, 9.237)	**<0.001**	2.665 (1.166, 6.087)	**0.020**
Hs-CTnI (>2,180 vs. ≤2,180 pg/ml）	3.918 (1.939, 7.914)	**<0.001**	3.007 (1.843, 4.776)	**0.006**
MYO (>50.75 vs. ≤50.75 ng/ml）	0.671 (0.236, 1.904)	0.453		
CK-MB（>17.91 vs. ≤17.91 U/L）	2.392 (1.272, 4.496)	**0.007**	0.890 (0.433, 1.831)	0.752
D-Dimer (mg/L)	1.115 (0.925, 1.345)	0.254		
FDP (ug/L)	1.059 (0.994, 1.129)	0.078		
HbA1C (%)	0.892 (0.691, 1.152)	0.382		
TG (mmol/L)	0.996 (0.810, 1.224)	0.967		
HDL-c (mmol/L)	0.615 (0.229, 1.655)	0.336		
LDL-c (mmol/L)	0.886 (0.679, 1.156)	0.373		
Lpa (mmol/L)	0.999 (0.998, 1.001)	0.500		
HCY (mmol/L)	1.016 (0.990, 1.043)	0.218		
LVEF (40%-49% vs. ≥50%)	7.532 (3.737, 15.181)	**<0.001**	3.590 (1.540, 8.370)	**0.003**
LVEF (<40% vs. ≥50%)	11.686 (5.654, 24.153)	**<0.001**	5.077 (1.921,13.423)	**0.001**
LVESV (male >62 vs. ≤62 ml; female >47 vs. ≤47 ml)	0.941 (0.130, 6.828)	0.952		
LVEDV (male >156 vs. ≤156 ml; female >121 vs. ≤121 ml)	2.577 (1.370, 4.847)	**0.003**	0.847 (0.377, 1.901)	0.687
LVDF (I vs. 0)	1.378 (0.509, 3.736)	0.528		
LVDF (II vs. 0)	2.041 (0.748, 5.572)	0.164		
LVDF (III vs. 0)	1.634 (0.518, 5.147)	0.402		
IVS (mm)	1.152 (0.959, 1.383)	0.131		
LVPW (mm)	1.064 (0.828, 1.367)	0.627		
No reflow Slow blood flow (yes vs. no)	1.673 (0.909, 3.081)	0.099		
Intraoperative hypotension (yes vs. no)	0.981 (0.415, 2.318)	0.966		
Intraoperative ventricular arrhythmia (yes vs. no)	1.184 (0.501, 2.797)	0.700		
TIMI classification (non-III vs. III)	1.184 (0.501, 2.797)	0.700		
SYNTAX score	0.961 (0.911, 1.014)	0.148		

EAMI, epicardial adipose tissue mass index; EATV, epicardial adipose tissue volume; EAAI, epicardial adipose tissue attenuation index; BMI, body mass index; NLR, Neutrophil-lymphocyte ratio; CRP, C-reactive protein; HGB, hemoglobin; PLT, platelet count; PDW, platelet distribution width; ALT, alanine aminotransferase; AST, aspartate aminotransferase; Cr, creatinine; TBIL, total bilirubin; UA,uric acid; NT-proBNP, N-terminal pro B-type natriuretic peptide; MYO, myoglobin; Hs-CTnI, high-sensitivity troponin I; CK-MB, creatine kinase MB; FDP, fibrinogen degradation product; HbA1c, glycosylated hemoglobin; TG, triglycerides; HDL-c, high-density lipoprotein cholesterol; LDL-c, low-density lipoprotein cholesterol; Lpa, lipoprotein a; HCY, homocysteine; LVEF, left ventricular ejection fraction; LVEDV, left ventricular end-diastolic volume; LVESV, left ventricular end-systolic volume, LVDF, left ventricular diastolic function; IVS, interventricular septal thickness; LVPW, left ventricular posterior wall thickness; TIMI, thrombolysis in myocardial infarction; SYNTAX, SYNergy between PCI with TAXUS™ and Cardiac Surgery.

Bold indicates statistical significance.

### Predictive performance of EAMI for MACE in training set

**Figure 5:**
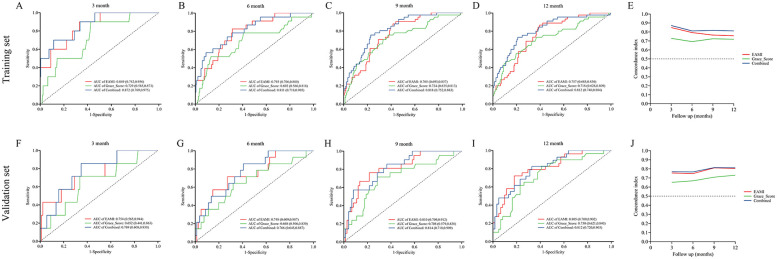
ROC curve and C-index for the prediction of MACE by EAMI and GRACE score at follow-up time, training set **(A–E)**, validation set **(F–J)**.

**Figure 6:**
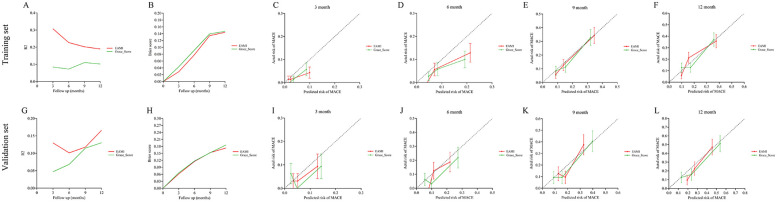
R^2^, brier score and calibration curve for the prediction of MACE by EAMI and GRACE score at follow-up time, training set **(A–F)**, validation set **(G–L)**.

**Figure 7:**
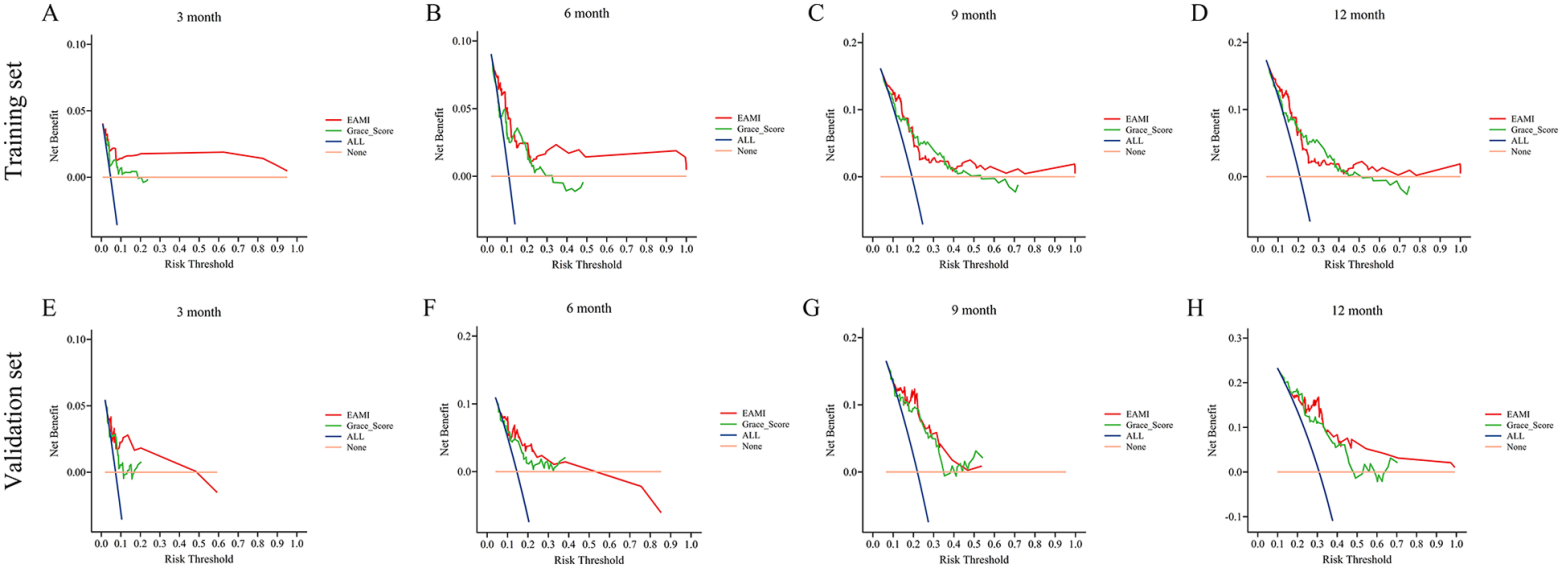
DCA for the prediction of MACE by EAMI and GRACE score at follow-up time, training set **(A–D)**, validation set **(E–H)**.

In training set, the ROC analysis (Figures 5A–D) indicated that EAMI has better predictive discrimination than GRACE score (AUC: 0.849 vs. 0.729, *p* = 0.022 at 3-month; AUC: 0.793 vs. 0.692, *p* = 0.025 at 6-month; AUC: 0.765 vs. 0.724, *p* = 0.032 at 9-month; AUC: 0.753 vs. 0.707, *p* = 0.030 at 12-month). The C-index (Figure 5E) further suggested that EAMI outperforms the GRACE score in predicting MACE, accounting for the time factor. The NRI (0.244, 95CI: 0.095–0.413) and IDI (0.197, 95CI: 0.094–0.332) show that EAMI has a positive improvement in predicting MACE compared to GRACE score. Additionally, the R^2^ values (Figure 6A) for EAMI at 3-, 6-, 9-, and 12-month follow-up were significantly higher than those for the GRACE score (0.307 vs. 0.085; 0.227 vs. 0.072; 0.202 vs. 0.111; 0.190 vs. 0.102, respectively), indicating a better model fit for EAMI. The Brier score (Figure 6B), which measures the accuracy of probability forecasts, was lower for EAMI than for the GRACE score at most time points (Brier: 0.029 vs. 0.043 at 3-month; 0.076 vs. 0.091 at 6-month; 0.124 vs. 0.139 at 9-month; 0.131 vs. 0.147 at 12-month). Calibration curves (Figures 6C–F) revealed superior predictive accuracy for EAMI relative to the GRACE score from 6 months onward. The decision curve (Figures 7A–D) showed that the clinical net benefit for EAMI exceeds that of the GRACE score at different follow-up periods across various threshold ranges (3-month: threshold range of 0.1–1.0; 6-month: threshold range of 0.2–1.0; 9-month: threshold range of 0.4–1.0; 12-month: threshold range of 0.45–1.0).

The C-index, R^2^, calibration curve, Brier score, and DCA together indicate that EAMI is a more reliable predictor of MACE in STEMI patients than its individual components, EATV or EAAI, alone (Supplementary Figures S1–S15).

### Predictive performance of EAMI for MACE in validation set

The results from the validation set were close to those from the training set. In validation set, ROC analysis (Figures 5F–I) shows that EAMI outperforms the GRACE score in predictive discrimination, with higher AUC values at 3-month (0.754 vs. 0.652, *p* = 0.023), 6-month (0.748 vs. 0.668, *p* = 0.034), 9-month (0.810 vs. 0.708, *p* = 0.020), and 12-month (0.805 vs. 0.730, *p* = 0.036). The C-index (Figure 5J) confirms that EAMI remains a more effective predictor of MACE over time. The NRI (0.179, 95CI: 0.078–0.280) and IDI (0.111, 95CI: 0.003–0.219), both showed EAMI increase the proportion of correct classification and improve the prediction effect than GRACE score. R^2^ values (Figure 6G) for EAMI are consistently higher than those for the GRACE score at all follow-up points: 3-month (0.130 vs. 0.047), 6-month (0.101 vs. 0.068), 9-month (0.118 vs. 0.115), and 12-month (0.166 vs. 0.130). The Brier score (Figure 6H) is lower for EAMI compared to the GRACE score at most time points: 3-month (0.062 vs. 0.066), 6-month (0.115 vs. 0.117), 9-month (0.153 vs. 0.153), and 12-month (0.173 vs. 0.186). Calibration curves (Figures 6I–L) demonstrate superior predictive accuracy for EAMI from 3 months onward. Decision curve analysis (Figures 7E–H) shows that EAMI provides a greater clinical net benefit than the GRACE score at various threshold ranges: 3-month (0.0–0.5), 6-month (0.0–0.5), 9-month (0.0–0.5), and 12-month (0.25–1.0).

### Predictive performance of EAMI combined with GRACE score

In training set, the ROC analysis (Figures 5A–D) indicated that EAMI + GRACE score has better predictive discrimination than individual GRACE score. At 3 months, AUC: 0.872 vs. 0.729, *p* = 0.012; At 6 months, AUC: 0.811 vs. 0.692, *p* = 0.002; At 9 months, AUC: 0.818 vs. 0.724, *p* = 0.003; At 12 months, AUC: 0.812 vs. 0.718, *p* = 0.002. In validation set, the ROC analysis (Figures 5F–I) also indicated that EAMI + GRACE score has better predictive discrimination after 6 months. At 3 months, AUC: 0.769 vs. 0.652, *p* = 0.195; At 6 months, AUC: 0.766 vs. 0.668, *p* = 0.104; At 9 months, AUC: 0.814 vs. 0.708, *p* = 0.021; At 12 months, AUC: 0.812 vs. 0.730, *p* = 0.035.

### Subgroup analysis

**Figure 8:**
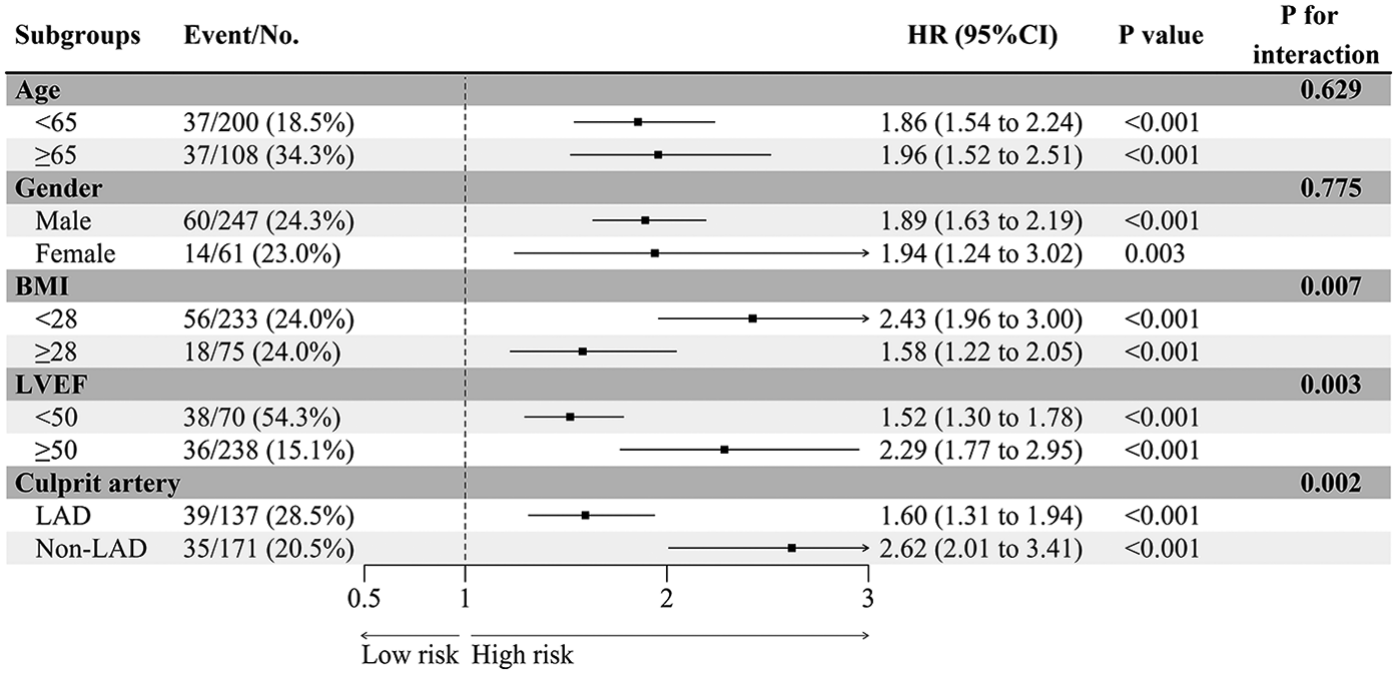
The HR of EAMI for MACE in different subgroups.

**Figure 9:**
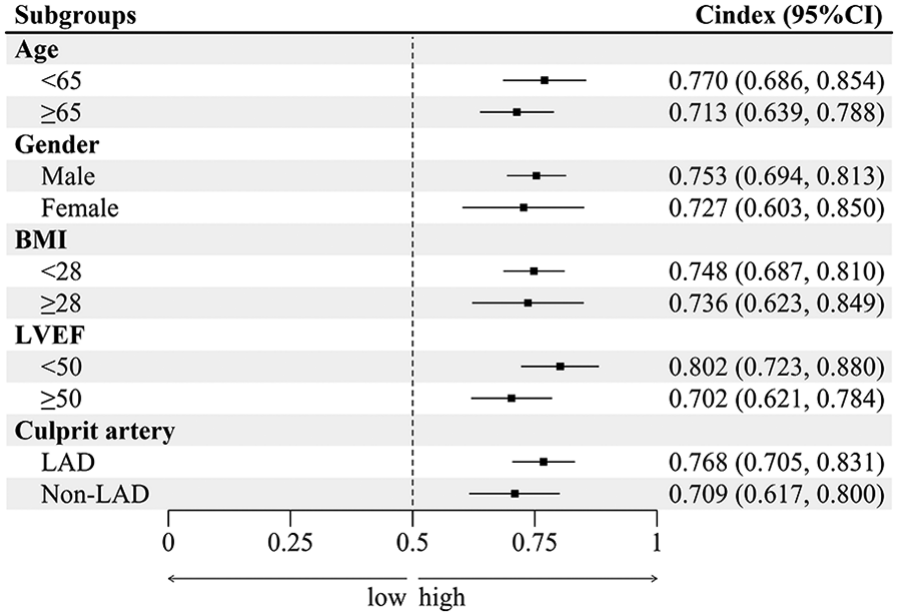
The C-index of EAMI for MACE in different subgroups.

EAMI is identified as an independent factor associated with MACE at one-year post-STEMI across various subgroups, including age, gender, BMI, LVEF, and culprit artery (Figure 8). Significant variations in the influence of EAMI on MACE are observed within the BMI, LVEF, and culprit artery subgroups (*P* interaction < 0.05). The C-index (Figure 9) demonstrates that, across all subgroups, the predictive discrimination of EAMI for MACE exceeds the 0.70 threshold.

## Discussion

This study demonstrates that EAMI, a novel index integrating both EAT volume and inflammatory response, is an effective predictor of MACE in STEMI patients. EAT has garnered increasing attention as a potential predictive and therapeutic target for various cardiovascular diseases. While EAT provides energy under normal conditions, its excessive accumulation exerts detrimental effects on the myocardium through the secretion of inflammatory mediators and cytokines (17). Gruzdeva et al. found that genes encoding inflammatory mediators, such as tumor necrosis factor-alpha, interleukin-6, and monocyte chemoattractant protein-1, were significantly upregulated in patients with excessive EAT deposition (18). Similarly, Huang et al. reported that abnormal EAT accumulation promotes macrophage polarization from a non-inflammatory M2 phenotype to a pro-inflammatory M1 phenotype via the NF-*κ*B pathway, leading to the secretion of inflammatory factors and reduced secretion of lipocalin and adrenomedullin (19). In rat models, Abe et al. demonstrated that EAT activates myofibroblasts and induces extracellular matrix deposition through the TGF-*β*/Smad pathway, ultimately contributing to interstitial fibrosis (20). Other studies have linked excessive EAT accumulation in the pericardial space with diastolic dysfunction in heart failure with preserved ejection fraction (21). Furthermore, EAT has been implicated in heart failure with reduced ejection fraction by inducing cardiomyocyte dysfunction, impairing oxidative phosphorylation, and promoting apoptosis (22). The pro-inflammatory, pro-fibrotic, and pro-apoptotic effects of EAT may contribute to post-STEMI ventricular remodeling, which underlies MACE. Additionally, inflammatory cytokines secreted by pericoronary EAT can directly damage vascular endothelial cells, thereby facilitating atheromatous plaque formation and increasing plaque vulnerability (23, 24). Gavara et al. confirmed through cardiac magnetic resonance imaging that higher EAT in STEMI patients often means larger IS, and high EAT and greater subsequent EAT reduction were linked to more preserved LVEF in the chronic phase (7). The dual and paradoxical effect of EAT in the conclusion of this study may be related to only considering EATV and ignoring the degree of inflammatory response in EAT. This also reflects the progressiveness of this study in evaluating EAT methods to some extent.

As is well known, obesity is one of the important factors contributing to poor prognosis in STEMI patients. Currently, the evaluation of obesity is becoming increasingly precise, from the systemic obesity index - BMI to the exclusive cardiac obesity index - EAT. West et al. pointed out EAT forms a powerful marker of metabolically unhealthy visceral obesity, which could be used for cardiovascular risk stratification (25). EAT may have already played a pathological role in the early stages of heart failure in obese patients (26). Wacker et al. found a good correlation between EAT and abdominal obesity in cardiovascular disease populations (27). However, previous studies have not fully demonstrated the value of EAT. To the best of our knowledge, this is the first study to introduce EAMI, a comprehensive imaging-based parameter, for predicting MACE in STEMI patients. EAMI integrates both EATV and EAAI, which represent the anatomical and biological effects of EAT, respectively. Our analysis found that systemic biomarkers such as BMI and C-reactive protein were not independently associated with MACE. Subsequent restricted cubic spline analysis revealed a dose-response relationship between EAMI and MACE in STEMI patients. Moreover, during the follow-up period, the AUC at different time points of EAMI was higher than the GRACE score, the difference was statistically significant (*p* < 0.05). Indicating that EAMI is indeed superior to GRACE score in terms of predictive discrimination ability. Previous studies, which have predominantly used EATV alone as an outcome measure, fail to account for EAT lipotoxicity. EAT lipotoxicity correlates with inflammatory processes within the EAT, which can be quantified using the EAAI on CT scans (20). Antonopoulos et al. found that a higher EAAI, partly due to immature adipocytes with reduced fat content, is associated with inflammatory cytokine activity (15). As shown in Supplementary Figures S10–S14, EAMI outperforms EATV or EAAI alone in predicting MACE outcomes in STEMI. Therefore, EAMI, which combines both the quantity and quality of EAT, may provide a more reliable prediction of MACE in STEMI than EAT volume alone.

The extended follow-up period and inclusion of additional endpoint events in this study enhance its robustness. Our findings indicate that EAMI offers superior predictive performance compared to the GRACE score across various follow-up periods. Adverse events post-STEMI are primarily driven by ventricular remodeling, which typically takes over six months to manifest structural alterations (28). Previous studies have criticized the GRACE score for its exclusion of NT-proBNP and the inadequate dichotomization of CTnI, which fails to capture the full extent of cardiac injury (29). Moreover, blood biomarkers are time-dependent and subject to confounding factors, such as therapeutic interventions, blood pressure, and heart rate, which can fluctuate significantly in the perioperative period. Killip grading, based on clinical examination, also introduces subjectivity and variability during this period (30). In contrast, EAT, as a stable human tissue, undergoes minimal changes in response to short-term perioperative interventions, making it a more suitable marker for early prediction of long-term outcomes. Although EAT is an extra-cardiac tissue, it directly affects the myocardium both anatomically and physiologically, reflecting long-term pathological impacts (31). EAMI, which balances EAT volume and inflammatory response, relies on CTA image post-processing software to measure adipose tissue density, offering a more objective and reliable methodology. From a mechanistic perspective, EAMI, as an imaging parameter, can supplement the GRACE score (which does not have imaging indicators) and to some extent compensate for the lack of stability in GRACE scoring items. In this study, the combination of EAMI and GRACE score showed better predictive discrimination than GRACE alone. This discovery provides new ideas for the clinical application of EAMI. It is also worth noting that EAT, beyond being a marker, represents a potential therapeutic target. Díaz-Rodríguez et al. demonstrated that dapagliflozin could provide myocardial protection by improving EAT cell differentiation (32). The EMPA-TROPISM study found that empagliflozin significantly reduced EAT volume compared to placebo (−5.14 ml vs. −0.75 ml, *P* < 0.05), improving clinical outcomes in HFrEF patients (33).

Subgroup analysis further supports the clinical utility of EAMI in predicting MACE. It showed that EAMI significantly impacts MACE across various subgroups, including age, gender, BMI, LVEF, and culprit artery. Notably, within the BMI, LVEF, and culprit artery subgroups, EAMI demonstrated significant differences in hazard ratios for MACE (*P* interaction < 0.05), suggesting that EAMI has a greater impact on individuals with normal BMI and is a more specific indicator of “cardiac obesity”. Additionally, EAMI showed high predictive power in the normal LVEF population, highlighting the importance of EAT in STEMI patients with preserved cardiac function. In the Non-LAD subgroup, EAMI exhibited stronger predictive power, likely because RCA and LCX, which supply the inferior and lateral walls of the left ventricle, have a smaller effect on cardiac function compared to the anterior wall supplied by the LAD. This finding aligns with previous studies showing a higher incidence of poor prognosis in LAD myocardial infarction (34). Thus, these results underscore the need to closely monitor EAT status in Non-LAD STEMI patients.

This study has several limitations. First, as a single center observational study, it cannot establish causal relationships and may suffer from selection bias. Second, this study did not include NSTEMI patients in the analysis. Compared to STEMI, NSTEMI often has characteristics such as multivessel disease and incomplete occlusion of culprit vessels, resulting in myocardial necrosis that is mostly limited to the subendocardial or smaller range. Third, there may be residual confounding factors that were not accounted for in the analysis. For example, lifestyle factors such as diet or physical activity, which could influence EAT accumulation and inflammatory responses, were not fully considered in this study, potentially affecting the observed relationship between EAMI and MACE. Finally, the predictive discrimination of EAMI was higher than the GRACE score during the 1-year follow-up period, but showed a decreasing trend. Further research is needed to verify whether EAMI can maintain outstanding predictive performance over a longer follow-up period.

## Conclusions

In conclusion, this study introduces a novel imaging-based parameter, EAMI, which effectively predicts MACE in STEMI patients. Notably, EAMI demonstrates superior predictive accuracy and clinical applicability compared to the traditional GRACE score. However, due to the single-center design of this study, further large-scale, multi-center research is needed to validate the prognostic value of EAMI in predicting MACE post-STEMI.

## Data Availability

The datasets presented in this study can be found in online repositories. The names of the repository/repositories and accession number(s) can be found in the article/Supplementary Material.
