# Supplementary figures and images for: Prediction of major adverse cardiovascular events following ST-segment elevation myocardial infarction using cardiac obesity marker—epicardial adipose tissue mass index: a prospective cohort study
Source: Front Cardiovasc Med. 2025 Feb 12;12:1539500. doi: 10.3389/fcvm.2025.1539500 (PMC11861378; doi:10.3389/fcvm.2025.1539500)

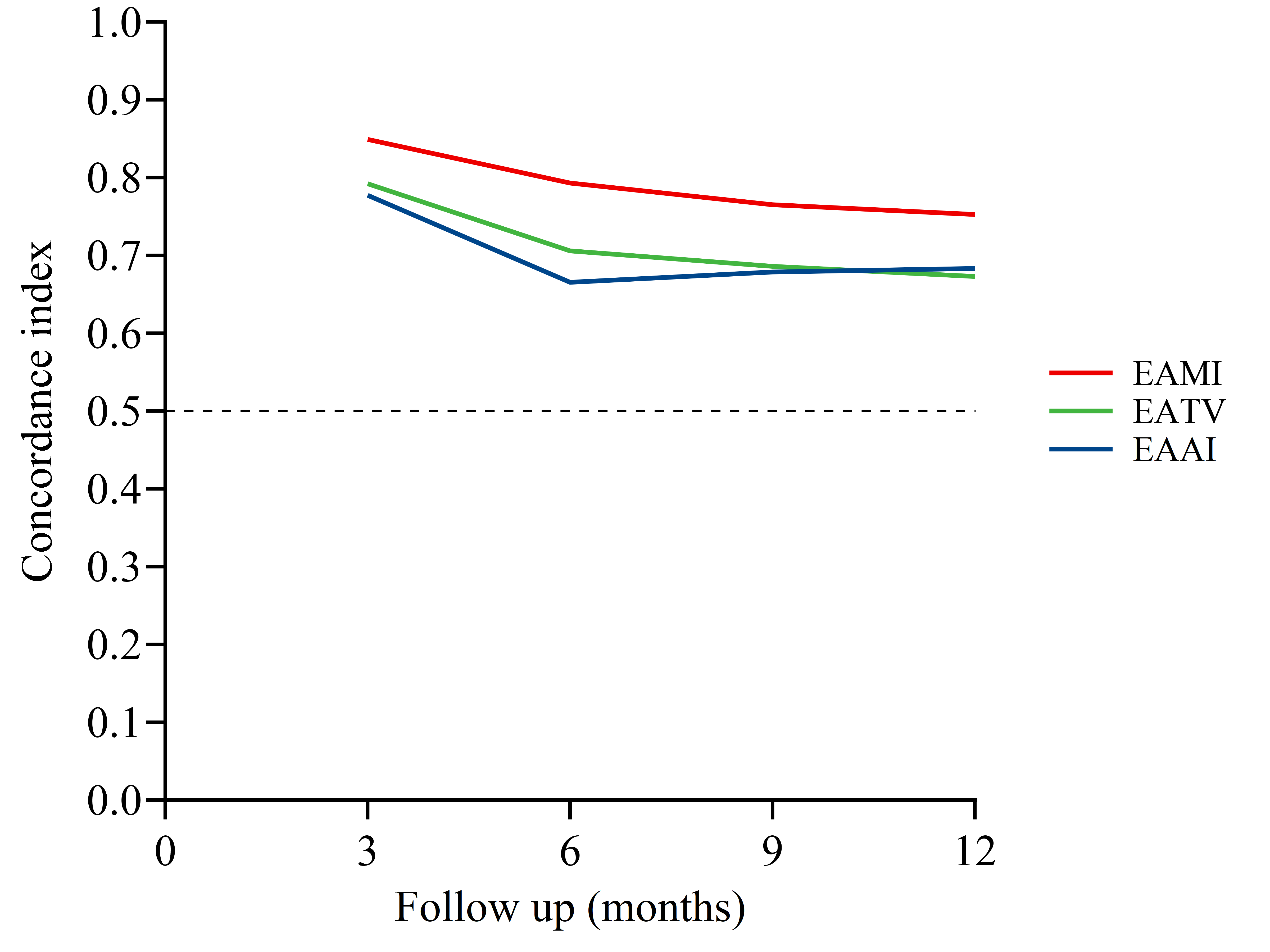

Supplement: Supplementary file 1 [file Image1.tif]

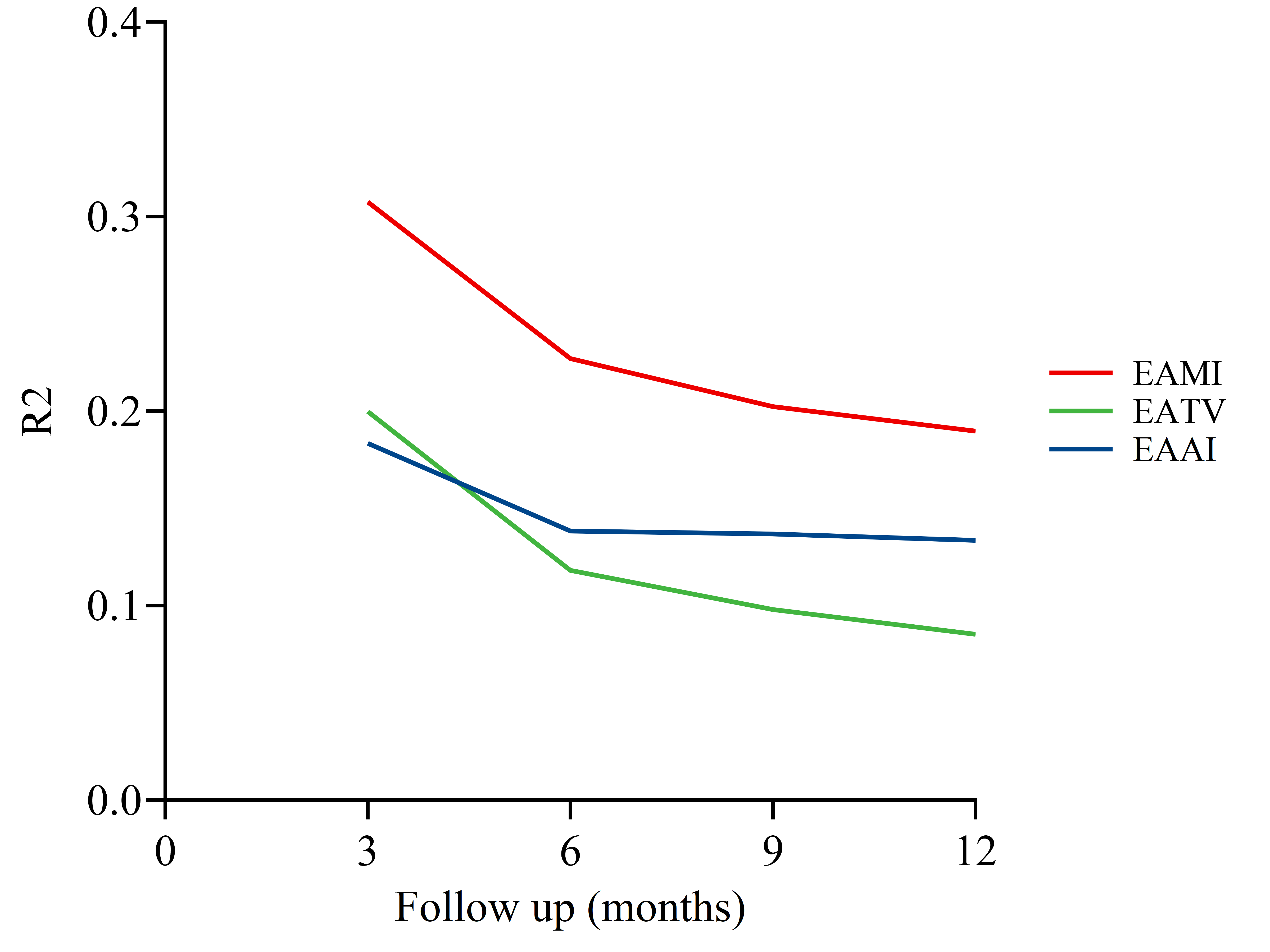

Supplement: Supplementary file 2 [file Image2.tif]

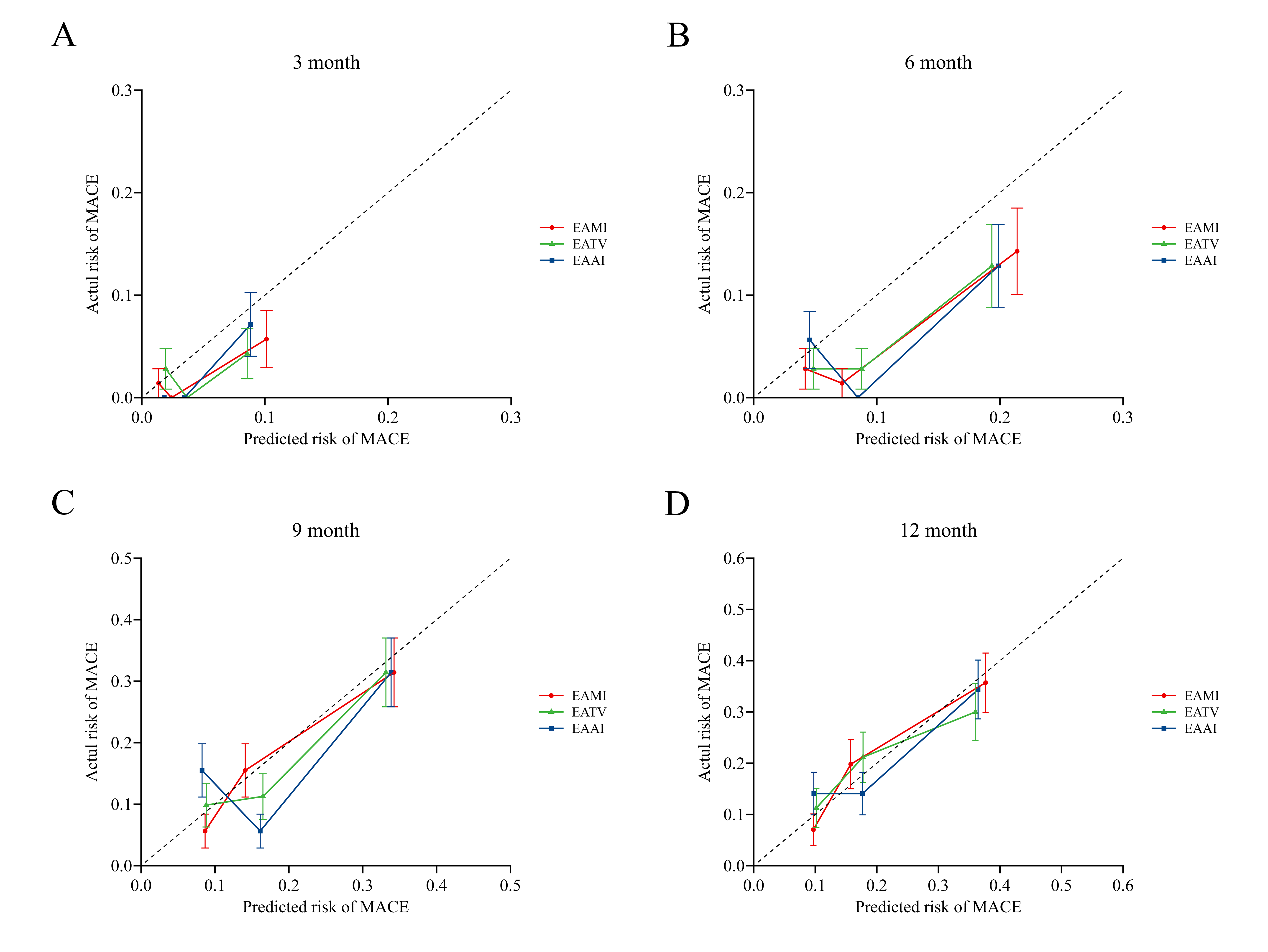

Supplement: Supplementary file 3 [file Image3.tif]

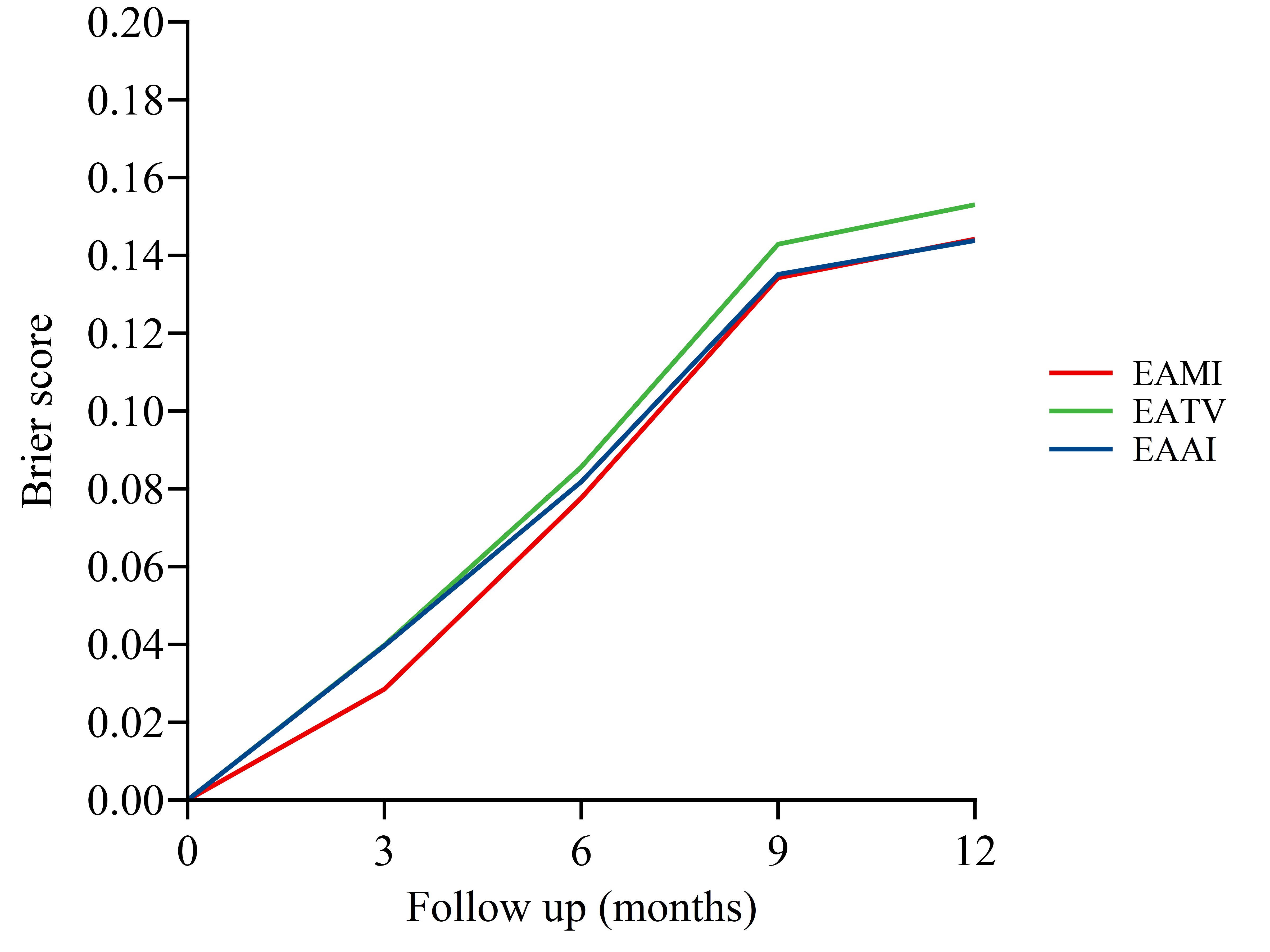

Supplement: Supplementary file 4 [file Image4.tif]

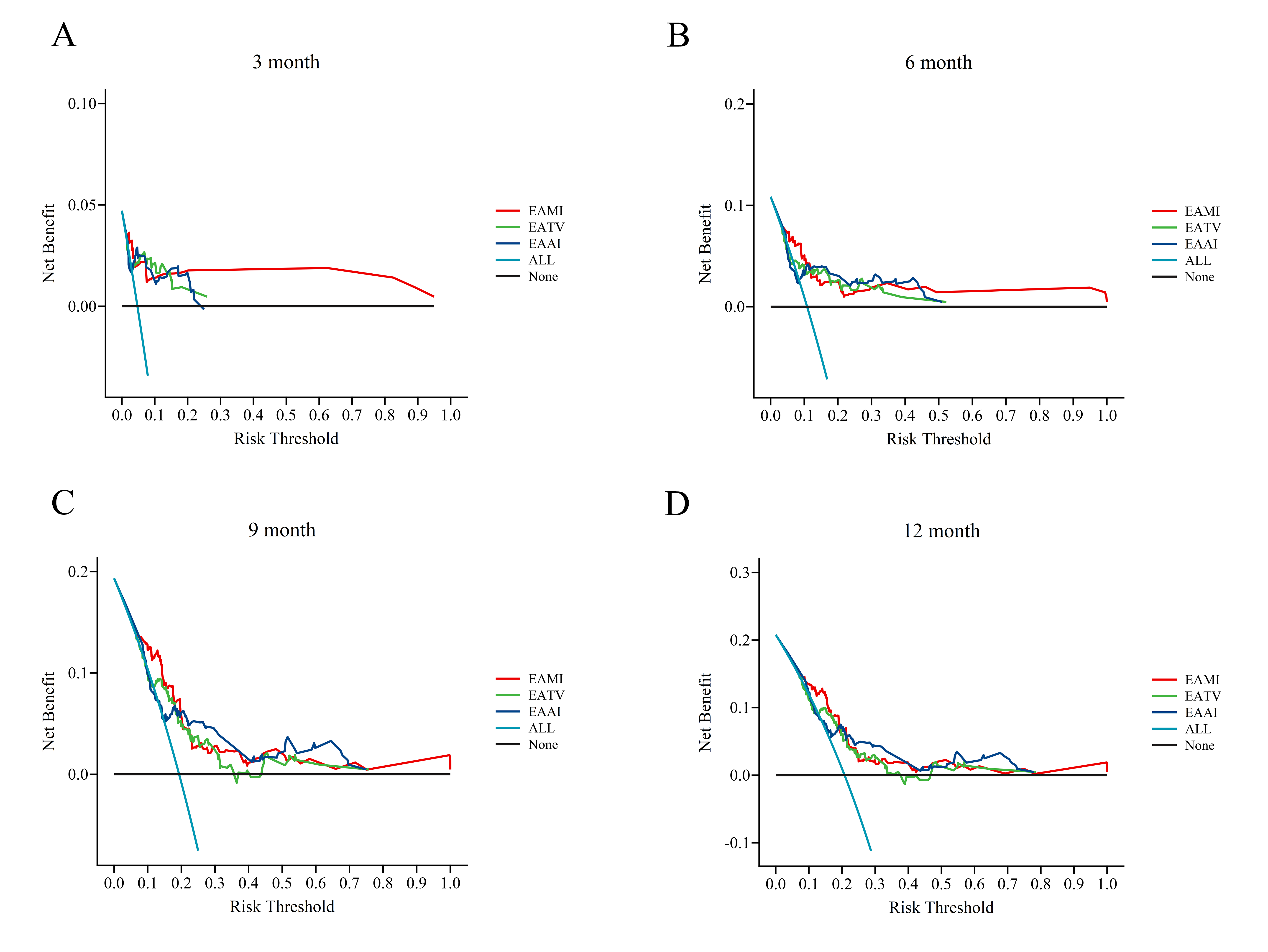

Supplement: Supplementary file 5 [file Image5.tif]
